# O.4.4-6 Preliminary research: exploring the barriers and motivators for women from an ethnic minority in South Cardiff taking part in walking and running groups

**DOI:** 10.1093/eurpub/ckad133.203

**Published:** 2023-09-11

**Authors:** Faaiza Bashir, Ellyse Hopkins

**Affiliations:** Cardiff Met University, UK; fbashir2@cardiffmet.ac.uk; Cardiff Met University, UK; fbashir2@cardiffmet.ac.uk

## Abstract

Physical activity (PA) levels are significantly lower in women from ethnic minorities and deprived areas compared to the general population throughout Cardiff owing to barriers affecting participation (Sport Wales, 2022). Given the far-ranging benefits of PA, there was a need to explore this relationship and promote opportunities for this population.The lead researchers were employees of Cardiff Met Sport (the city's sport development body) and had stakeholder good links to develop this project.

Our work specifically relates to

- Healthy Wales Healthy Weight - Welsh Government's long-term obesity strategy.

- One of the 7 goals in the Well-being of Future Generations Act (2015) Wales (i.e. 'A Healthier Wales').

- The Cardiff and Vale University Health Board's Physical Activity and Health Strategy that aims to reduce inequality through health (https://www.movemoreeatwell.co.uk).

The target audience for this work were:

a) Walk/run leaders/physical activity providers and community groups that specifically support women from ethnic minorities (n = 10). Our focus group explored the how organisations promote diversity in their activities, and the barriers in increasing community partcipation in PA. We also talked about co-producing a walking/running pilot in the area and invited people to take part. The key themese were trust-building, culturally sensitive activities and how resource-deprivation affects women.

b) Adult women from ethnic minorities (n = 18). We targeted populations living in South Cardiff, Wales, UK an area of deprivation. We ran 3 in-person focus groups to understand experiences of women, and the motivators and barriers to taking part in PA. Caring roles acted as a barrier and shortage of women-only spaces were highlighted. Women were active in community run activites and keen to be active.

We shared knowledge amongst walk/run leaders via e-mail and will organise a webinar to outline our findings, but are still finalising anlysis of this phase. We're developing the work by running a pilot intervention in Spring 2023.

Our participatory research has shown how to overcome the challenges of recruitment in this population. Our findings have implications for sport delivery patners (e.g. PA needs to be culturally-sensitive) and more widely shows that PA promotion cannot be separated from racial/political issues.

